# The emergency medical services network’s response to the COVID-19 pandemic in Albania

**DOI:** 10.3389/fpubh.2025.1568639

**Published:** 2025-07-30

**Authors:** Niccolò Persiani, Martina Giusti, Francesco Taiti, Andrea Biancalani, Michele De Luca, Maria José Caldés Pinilla

**Affiliations:** ^1^Department of Experimental and Clinical Medicine, University of Florence, Florence, Italy; ^2^Centro Studi SAPIS Foundation, Italian National Federation of Orders of Radiographers and Technical, Rehabilitation, and Prevention Health Professions Research Centre, Rome, Italy; ^3^IRCCS University Children Hospital Meyer, Florence, Italy; ^4^Department of Health, Tuscany Region, Florence, Italy; ^5^Global Health Centre, Tuscany Region, Florence, Italy

**Keywords:** COVID-19, emergency medical services, assessment, health emergency strategies, transition countries

## Abstract

**Background:**

During the COVID-19 pandemic, healthcare systems worldwide have implemented many health emergency plans to address the crisis. Following initial predominantly hospital-centred approaches, community-based healthcare assistance emerged as a more effective response to the emerging population needs. In low-middle-income countries, and particular in the so-called transition countries, the adaption the complexities of integrating pre-hospital and in-hospital Emergency Medical Services (EMSs) have been particularly challenging due to the absence of a consolidated network among these services. This research aimed to evaluate the emergency healthcare services response to covid-19 pandemic in Albania, as significant transition country.

**Method:**

The country case study methodology was deemed the most fitting approach for this research. Albania was selected as a notable case study due to its continuous endeavours towards achieving national welfare aligned with European standards, especially in the healthcare sector, as it has been moving towards pre-adhesion to the European Union.

**Results:**

Albanian EMSs network demonstrated its capability to update over time the national strategical plan against COVID-19 pandemic according to emerging evidence and the related organizational issues to effectively satisfy population health needs. This adaptability became feasible with the introduction of a modern EMSs system, comprising both pre-hospital and in-hospital dimensions. These two components collaborated and are still collaborating to implement integrated healthcare pathways, each with distinct responsibilities, resources, and protocols.

**Conclusion:**

The development, consolidation, and collaboration between pre-hospital and in-hospital EMSs implemented in Albania have played a crucial role in preventing the collapse of the healthcare system in the face of the COVID-19 pandemic. Albanian experience provides valuable insights for the reform or to build up EMSs network and healthcare systems in transition countries, drawing upon the lessons learned from the challenges posed by the COVID-19 pandemic.

## Highlights

- During the COVID-19 pandemic, the Albanian Emergency Medical Services network demonstrated its ability to adapt to emerging evidence and organizational challenges, effectively addressing the health needs of the population.
- The introduction of a modern EMS network—integrating both pre-hospital and in-hospital services—has facilitated more efficient reorganizations within the national healthcare system.
- The development, consolidation, and coordination between pre-hospital and in-hospital emergency services in transitioning countries played a vital role in preventing the collapse of national healthcare systems during the pandemic crisis.

## 1 Introduction

The COVID-19 pandemic emerged as the most significant global health crisis of recent decades, leaving a profound and lasting impact on societies worldwide. Devastating effects can largely be attributed to the world’s lack of preparedness in facing a health emergency of such magnitude, despite the existing of extensive warning on such risks (1, 2). Previous events—such as terrorist attacks of September 11 (3, 4), infective disease outbreaks like H1N1 influenza (5, 6) and Ebola (7, 8), as well as climate change-related disasters (e.g., floods, desertification, landslides, rising sea levels) (9, 10)—had already highlighted the need for robust emergency preparedness. Nevertheless, only a limited number of countries had developed comprehensive health emergency plans capable of rapidly reorganizing healthcare services and implementing timely interventions.

In the absence of coordinated international strategies, each country was compelled to independently devise its own containment measures during the initial wave of the COVID-19 pandemic, based on the structure and capacity of its national public health system (11). High-income countries, characterized by well-resources and high-performing healthcare systems (12), were generally better equipped to respond, with the ability to reallocate substantial resources to emergency departments and critical care settings (13). In contrast, low-and middle-income countries (LMICs) faced the pandemic with limited economic, organizational, and managerial resources (14–16). A key vulnerability in LMICs was the lack of integrated care networks, including essential services such as coordinated emergency medical systems (17). This structural weakness hindered the effective implementation of prevention, management, and intervention strategies, contributing to a higher incidence of epidemics, endemics, and pandemics compared to high-income countries (18, 19).

Among LMICs, transition countries hold particular relevance. A transition country (or transitioning country) refers to a nation undergoing a process of significant political, economic, and social transformation—typically moving from a centrally planned economy (often under authoritarian or socialist regimes) towards a market-oriented economy and democratic governance. Key Characteristics of Transition Countries are economic reform, political change, social transformation, and institutional development. Many countries in Eastern Europe, the Balkans, and the former Soviet Union (e.g., Albania, Ukraine, Georgia, Kazakhstan) are considered transition countries. Some Asian and African nations undergoing democratization and economic liberalization may also be included, depending on context (20, 21).

In recent years, many of these nations have undergone comprehensive reforms, often driven by democratic transitions and supported by sustained economic growth. These reforms have prioritized the development of welfare state systems, with healthcare emerging as a central focus (22).

The overarching goal has been to align national health indicators with those of high-income countries, emphasizing the quality and equity of healthcare services in pursuit of universal health coverage—one of the United Nations’ Sustainable Development Goals (SDG 3) (23). In the context of emergency medical services (EMS), LMICs often lack adequate emergency departments capable of delivering complex care beyond basic first aid. In transition countries, while both pre-hospital and in-hospital EMS are generally available, a persistent challenge remains the lack of integration between these services (24, 25). Consequently, recent healthcare reforms in these countries have increasingly focused on developing effective EMSs networks. The establishment of such networks has a transformative impact on the social fabric, benefiting urban centres, suburban areas, and rural communities alike. Albania serves as a pertinent example. Following a prolonged transition from a Soviet-style totalitarian regime, the country has experienced significant economic development (26). This growth has enabled the government to initiate reforms aimed at aligning public services with European standards (27–30), with the broader objective of meeting the European Union’s accession criteria (31).

As other transition countries, Albania has sought in healthcare to overcome the legacy of the Semashko model inherited from the Communist era (26, 32). A major milestone was the launch of the national EMS network in 2018, centred around the National Emergency Centre 127, accessible via a dedicated national emergency number. This centre coordinates pre-hospital EMS across the country and maintains communication with 28 emergency departments distributed across districts (12), regions (11), and university hospitals (5). International cooperation has played a key role in supporting Albania’s efforts through capacity-building initiatives and training programs, contributing to policy reform and yielding not only economic but also significant social benefits (33–36).

Despite these advancements, challenges remain—particularly in ensuring full territorial coverage (37).

While several studies have examined the impact of the COVID-19 on healthcare systems in LMICs, they have often only indirectly addressed the consequences of inadequate EMSs network. These studies have primarily focused on the reduction in critical care delivery (38–40), delays in time-sensitive procedures (41, 42), and increased stress on emergency departments personnel (43, 44). However, there is a notable lack of research specifically examining the pandemic’s impact on EMSs in transition countries.

This study seeks to address a critical gap in the literature by examining the impact of evolving healthcare strategies during the COVID-19 pandemic on both pre-hospital and in-hospital EMSs in transition countries. Using Albania as a focal case study, the research aims to analyse systemic adaptations, operational challenges, and strategic responses within the EMSs network. The overarching goal is to contribute to the ongoing reorganization and modernization of Albania’s EMSs network and broader healthcare infrastructure.

Through evidence-based analysis, the study aspires to support policymakers, healthcare administrators, and emergency service providers in implementing sustainable reforms that improve service efficiency, responsiveness, and patient outcomes. By doing so, it intends to generate actionable insights and propose targeted recommendations for optimizing EMSs delivery and network and enhancing strategic resilience in similar healthcare contexts.

## 2 Method

### 2.1 Research design and case study selection

The country case study methodology (45) was identified as the most appropriate approach for this research due to its exploratory nature. Given the novelty of examining the impact of the COVID-19 pandemic on Emergency Medical Services (EMS) strategies in transition countries, a qualitative research design was essential to gain in-depth insights into this complex and underexplored topic (46, 47). While the case study method inherently presents limitations in terms of generalizability, the potential for analytical inference was enhanced by selecting a significant and representative case (48). Albania emerged as a compelling case study for several reasons. As part of its pre-accession process to the European Union, the country has been actively reforming its healthcare system to align with European health standards (49). Furthermore, the organizational structure of Albania’s healthcare system offered a clear framework for assessing the impact of COVID-19 across various levels of EMS—both pre-hospital and in-hospital—as well as within local health facilities during different phases of the pandemic. Notably, in 2018, Albania established a modern National Health Emergency Operative Centre, known as the National Emergency Centre 127, as part of a broader initiative to strengthen its welfare state infrastructure (50).

### 2.2 Data collection and analysis

The initial phase of developing the country case study involved designing a research protocol to ensure methodological consistency throughout the study (47, 51). This protocol detailed the data sources, identified key individuals for interviews, and outlined the specific questions to be addressed. The case study was conducted by clearly defining the field of investigation, namely the Emergency Medical Services (EMS) and their integration within the broader Albanian healthcare system. Data collection drew upon both primary and secondary sources. Primary data were gathered through semi-structured interviews, aimed at capturing insights into the evolving health strategies adopted by the Albanian government in response to the COVID-19 pandemic. The focus was placed on understanding the managerial, organizational, financial, and accountability-related dynamics between pre-hospital and in-hospital EMSs. A tailored interview guide was developed specifically for this study to support data collection. The full guide is provided in Supplementary file 1.

The interview questions were structured around three main areas of inquiry:

1. Description of Health Strategies. This section explored the various health strategies implemented by the Albanian government over time to manage the COVID-19 pandemic, with particular attention to the initial outbreak in 2020 and the second wave in 2021.
2. Evolution of the Organizational Model. This section investigated the development of the current organizational model of the Albanian EMS network, focusing specifically on the relationship between the pre-hospital component (National Emergency Centre 127) and the in-hospital component (emergency departments of district, regional, and university hospitals).
3. Consequences of Health Strategies. This section examined the outcomes of the different healthcare strategies adopted during the pandemic. It addressed aspects such as organizational restructuring, the roles assumed by various actors, and the inter-organizational relationships within the EMSs network. The analysis also considered procedural dynamics, organizational model implications, and supply-side factors.

10 face-to-face interviews were conducted with both medical and administrative representatives from each of the key organizations involved. Participants included managers and staff from the National Emergency Centre 127 (2 persons), representatives from the emergency departments and cost accounting offices of the Pogradec District Hospital (2 persons), the Vlora Regional Hospital (2 persons), and the Mother Teresa University Hospital in Tirana (2 persons), as well as management personnel from the National Health Insurance Fund (FSKDSH) (2 persons).

These five organizations were purposefully selected as they represent different levels within the Albanian Emergency Medical Services (EMSs) network:

- National Emergency Centre 127 – serving as the national coordinator among various EMS providers.
- Pogradec District Hospital – representing first-level EMS provision.
- Vlora Regional Hospital – representing second-level EMS provision.
- Mother Teresa University Hospital in Tirana – representing third-level EMS provision.
- National Health Insurance Fund (FSKDSH) – acting as the primary funder of EMS providers.

This manuscript is not the result of biomedical research, but rather a study conducted in the field of social sciences. Interviewees were selected exclusively for their managerial professional experience and their then-current role in each organization employed in the considered case study Therefore, ethics approval and informed consent to participate were not deemed necessary. However, consent for data management was obtained from the involved professionals, in accordance with current European privacy regulations (Regulation (EU) 2016/679-GDPR) and national legislation (Italian Legislative Decree 196/2003, “Personal Data Protection Code”).

The interview structure remained consistent across different managers to understand the different approaches and allow for effective comparison of the information obtained.

Semi-structured interviews were conducted in person, using a researcher-generated interview guide containing open-ended questions. The interviews were recorded and transcribed verbatim previous consent by interviewees. Below the questions were reported:

1. Could you please introduce yourself, including your profession and current role?
2. Which organization(s) are you currently working with? Could you describe the organization’s development and evolution over time?
3. Can you describe the different health strategies adopted by the Albanian government for managing the COVID-19 pandemic, particularly during the initial outbreak in 2020 and the subsequent waves in 2021?
4. What was your organization’s role in managing the initial impact of the COVID-19 pandemic on the Albanian healthcare system in 2020?
5. What role did your organization play during the subsequent waves of the COVID-19 pandemic in 2021?
6. Were any internal reorganizations implemented within your organization to align with the new health strategies adopted for managing the COVID-19 pandemic? If so, could you describe them?
7. How would you describe the collaboration and relationships with other organizations within the Albanian Emergency Medical Services (EMS) network during the pandemic?

The interviews were subjected to coding (52). The two main branches for coding analysis were “pre-hospitals” and “in-hospital” EMSs, because all interviewees have direct or indirect experience in the EMSs delivery.

Secondary data on both the quality (e.g., number of missions and medical consultations) and quantity of services provided by the National Emergency Centre 127 were obtained from the organization’s Statistics Department and verified in collaboration with involved local health facilities. Additionally, the National Health Insurance Fund (FSKDSH) provided data on the volume of hospitalizations and detailed economic information related to the emergency departments of all districts, regional, and university hospitals. These economic data included both direct costs—such as expenditures on pharmaceuticals and medical supplies, diagnostic kits and films, blood transfusions, food services, gross wages, and social security contributions—and indirect costs. Finally, secondary sources were also used to corroborate the information gathered through interviews and to enhance the overall validity and reliability of the findings (53).

## 3 Results

### 3.1 Pre-hospital emergency medical services in Albania: the National Emergency Centre 127

The implementation of Law 147/2014 marked the establishment of the National Emergency Centre 127, hereafter referred to as Centre. This Centre is tasked with coordinating medical transports, including ambulances and helicopters, for both emergency situations and secondary transfers among various health facilities. Centre 127 plays a crucial role in providing pre-hospital EMSs across the territory as part of the Albanian healthcare system.

In March 2015, the Centre was established to coordinate the public health transfers through a comprehensive review of pre-hospital EMSs system in Albania. Before each city had its own reference number to contact the local service for medical transfers, which delivered outpatient health transfers, emergency health transfers to emergency departments and transfers between different health facilities. There were no obligations for these services to respond to calls or provide the necessary services after a specific request. Moreover, no organization supervised on the activities carried out by the same (50).

In 2016, the Centre recruited personnel and bought the sufficient number of ambulances to serve the metropolitan area of Tirana. However, only in 2017 the Centre was fully operative in Tirana, following the implementation of the informative system to manage, monitor, and control Centre’s activities in real-time. In the same year, a promotional campaign was launched in the metropolitan area of Tirana to grow the knowledge of the Centre by the population, clarifying distinctions from the national emergency number “112” and aiming to prevent inadvertent mistakes in calling. Positive result of the effectiveness of this campaign is +395% calls to “127” of from April to December 2017.

In 2018, the Centre expanded its field of action nationwide, instituting approximately 440 ambulance stations near both hospitals and polyclinics. Another promotional campaign was launched to grow the knowledge of the Centre by the population at the national level. This campaign had also second purpose, the presentation of the entire EMSs system, both pre-hospital and in-hospital, to dispel the misconception that the provision of healthcare services of quality care was exclusively available in university hospitals located in the capital. The overcrowding of in university hospitals’ emergency departments by people arriving from the entire country was a critical issue due to the related organizational problems and the inappropriate accesses of low complexity that could be managed by regional hospitals.

In 2019, ambulance routes were optimized with the installation of GPS in all vehicles with direct consequences in terms of coordination of interventions and resources saving. In the same year, the intervention time was further reduced by equipping each ambulance with company mobile phones. These enhancements enabled Centre 127 to adopt standardized intervention procedures, strengthening coordination between different mobile units and enhancing overall performance.

In 2020 the Centre assumed with the onset of the COVID-19 pandemic in the role of the population’s reference centre as enacted by Albanian government. Operating 24/24 h, 7/7 days the Centre monitored the number of tests conducted and coordinated the reporting on the status of COVID-19 positive patients for the Albanian Ministry of Health.

In 2021 and 2022 the Centre demonstrated the capacity to manage the pandemic COVID-19 pandemic in all the different phases. In fact, this organization showed a high degree of adaptability reforming over time the own procedures in the management of home-case assistance as transfers of positive patients exclusively to university hospitals’ emergency departments in 2020 to the entire network of health emergency departments, including regional hospitals.

Up to date, the Centre is widely recognized by the Albanian population as the reference organization for health transfers, both in emergency and planned, at national level. The Centre has, in fact, a consolidated organizational model to provide pre-hospital EMSs, including missions, medical consultations, and medical transports within the Albanian healthcare system for internal calls. When the Centre receives a call, the own nurses and doctors promptly respond from centralized operative centrals to assess the situation presented by the caller and activate the most appropriate EMSs in relation to the patient’s health conditions. All calls, medical consultations, and completed transports are meticulously recorded in the before mentioned informatic system that supports the coding of the type of health emergency, attributing severity, and facilitating the provision of the most suitable EMS.

Ongoing developments for this service include ensuring radio coverage for all ambulances and implementing a backup system for the current informatic system, which is still absent. It should enable the continued operation of Centre even in critical contexts such as earthquakes or terrorist attacks and the strengthening of data collection culture into the organization. These interventions align with the implementation of the National Health Emergency Plan outlined in the Civil Protection reform law (Law 47/2019). The reassessment of the currently services delivered by the Centre is necessary for identifying eventual resources shortage and then, proceeding with the request of additions funding from the Ministry of Health to implement the National Health Emergency. Currently, the Albanian Ministry of Health finances the annual activities of Centre based on the implementation of a predefined annual programme that includes specific objectives proposed, discussed, and approved by both parts. In the next years, nevertheless, the development of multi-year plans are necessary to implement strategies for the enforcement of Albanian both pre-hospitals and in-hospitals EMSs, emphasizing the need for a comprehensive approach to ensure the continued success of National Emergency Centre 127.

### 3.2 Intra-hospital emergency medical services in Albania

Regarding in-hospital EMSs, the current infrastructure of Albanian healthcare system comprises 28 public first-aid points strategically located throughout the country and organized in three distinct levels of specialization.

The in-hospital EMSs network includes:

- *District Hospitals:* These facilities serve as first-aid points primarily for very low and low complexity cases. Structured with a minimum of four specialized departments—internal medicine, paediatrics, general surgery, and obstetrics/gynaecology—district hospitals provide inpatient care within their territories. Some are additionally entrusted with emergency services, anaesthesia, intensive care, radiology, biochemical laboratories, and service pharmacies.
- *Regional Hospitals:* Equipped with first-level emergency rooms, regional hospitals cater to medium to high complexity cases. With a range of 10 to 12 specialist departments, these hospitals ensure services with a higher degree of specialization compared to district hospitals.
- *University Hospitals:* These hospitals feature second-level emergency rooms and are exclusively situated in Tirana and Durres. Distinguished by their advanced medical capabilities, University Hospitals house departments such as cardiac surgery and neurosurgery, which are typically not available in regional hospitals.

Between 2009 and 2013, the restriction of access to medical specializations resulted in a severe shortage of healthcare professionals, leaving various departments, particularly emergency departments in suburban areas, that were really understaffed. Although there has been partial improvement with the reopening of bachelor’s and master’s degree programs in the health sector, the challenge of attracting medical professionals to the capital, Tirana, persists (54).

Nowadays, hospital departments receive patients through direct access or transports via ambulances or helicopters coordinated by the Centre (since 2017) or private medical transport services at national level. The admission process still lacks formalized triage system with the relates opening of a personal medical file for each patient. This absence hinders the implementation of control mechanisms on every type of activity done in these departments. Consequently, only the overheads of these departments provide partial figures on their activity volumes.

As integral components of the Albanian healthcare system, the network of public emergency departments is funded on historical expenditure by the National Health Insurance Fund (FSKDSH).

### 3.3 The emergency medical services network in Albania

Up to date, the Albania EMSs network is managed by the National Emergency Centre 127, that is entrusted with two core responsibilities:

- The provision of pre-hospital EMS across the entire national territory, responding to calls made to the national health emergency number “127.”
- The coordination of in-hospital EMS, delivered through a network of 28 emergency departments, distributed among twelve district hospitals, eleven regional hospitals, and five university hospitals.

Despite the efforts of the Albanian Ministry of Health to strengthen its capacity for managing health emergencies, several critical areas still require improvement (37). The reason is that pre-hospital and in-hospital EMSs have continuing to operate in a fragmented manner due to various systemic and organizational challenges.

One major issue is the lack of a shared informatic system. All ambulances of National Emergency Centre are equipped with a digital system that manages interventions, collects patient anamnesis, records intervention details, verifies adherence to established protocols, and transmits real-time data to centralized control centres. On the base of received information, control centres determine whether a hospital transfer is necessary or not.

Because the same information system is not adopted by hospital emergency departments, a new triage process is essentially carried out upon the patient’s arrival at the hospital. In fact, it is no mandatory to share the initial patient assessment conducted by National Emergency Centre personnel with hospital emergency department staff. Although ambulance teams currently provide a paper-based report summarizing the interventions already performed—intended to prevent conflicting treatments (e.g., duplicate drug administration)—but this document does not serve as a formal starting point for hospital-based care. Moreover, no integrated clinical pathways have been developed to coordinate care across all levels of in-hospital EMS undermining continuity of care and limiting collaborative decision-making between pre-hospital and in-hospital EMSs’ providers.

Another major issue is the absence of an implementation plan for the Albanian EMS network, which determined the imposition to transfer patients who required in-hospital EMSs, exclusively to the reference regional hospital in relation to the place of intervention, regardless of the patient’s clinical condition. In this way, district-level hospitals are excluded to the EMSs network also if they have first aid services. Furthermore, the no pre-arrival communication to destination hospitals with all related problems in terms of overbooking, unpreparedness, the lack respect of time-sensitive procedures and so on.

Another critical issue concerns the heterogeneity in the availability and coverage of EMSs across the country. In particular, there are still regions—especially those geographically distant from the capital, Tirana—that are either not served at all by the medical transport services provided by the National Emergency Centre, or where the 127 service is not yet operational. In fact, the division of the entire national territory in the area of intervention for each individual mobile unit is still underway, but there are still not enough ambulances to guarantee the service. The expansion of the equipment park is therefore underway. This uneven distribution of services has significant implications for equity in access to both pre-hospital and in-hospital emergency care. Here, transport is therefore still the prerogative of citizens. So, populations residing in these underserved areas face delays in receiving timely medical assistance, which can lead to worsened health outcomes and increased mortality risk in emergency situations. Moreover, the lack of standardized service provision across regions contributes to disparities in the quality of care, undermining the principles of universal health coverage and equal treatment regardless of geographic location.

**Figure 1:**
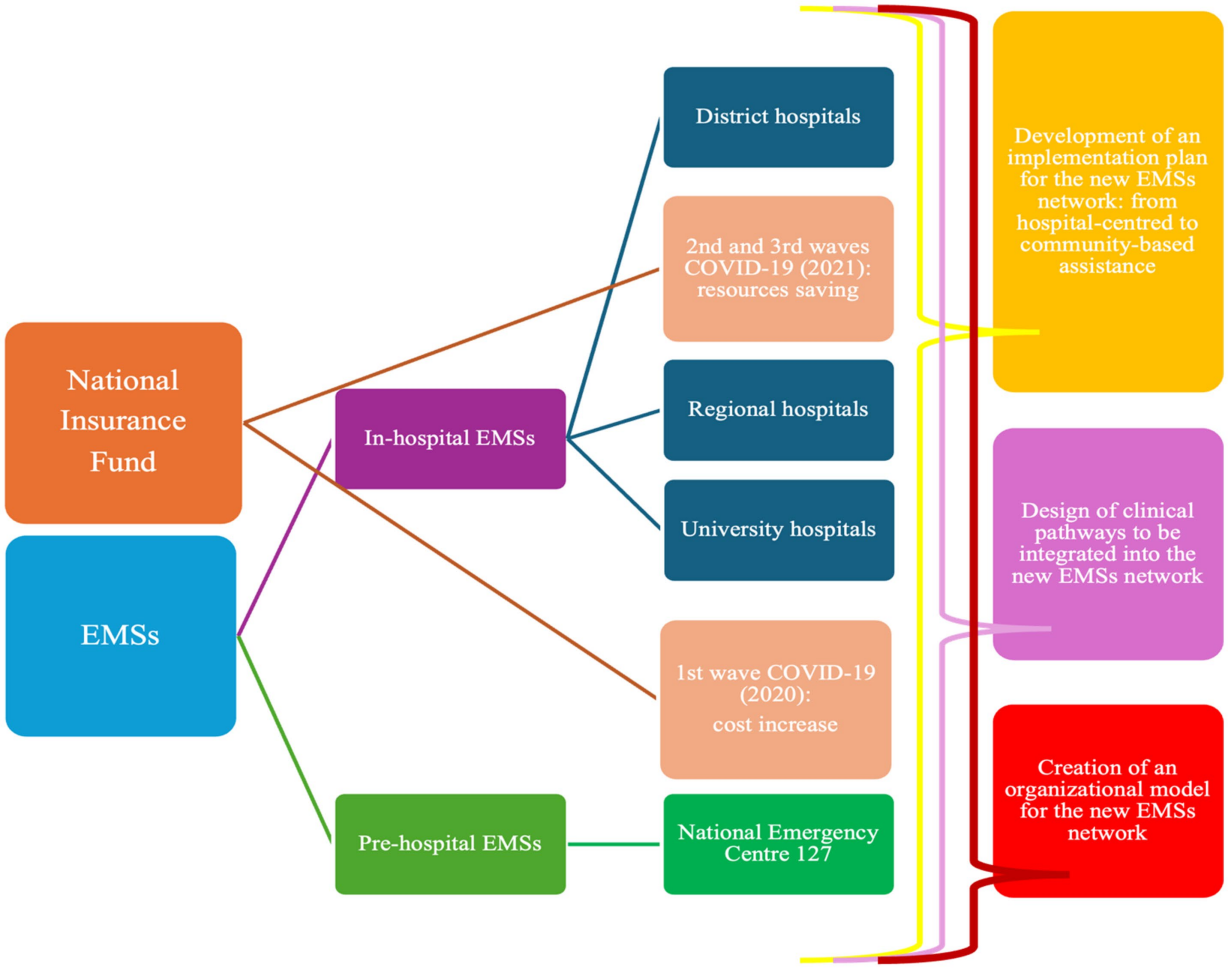
Coding tree resulted by the interviews’ analysis.

For these reasons, all interviewees consistently emphasized the same key priorities for the future development of the Albanian EMS network:

- The development of a comprehensive implementation plan for the EMSs network.
- The design of integrated clinical pathways to be applied across all EMSs components.
- The creation of a coherent organizational model for the national EMSs system (Figure 1).

Due these issues, Albania is actively working towards achieving universal health coverage in alignment with the target 3.8 of the 3rd SDG. Investments in health emergency capacities contribute significantly to the country’s progress, particularly in realizing the 3rd Sustainable Development Goal “Good health and well-being.” Universal health coverage ensures that people can access high-quality, affordable, safe, and culturally sensitive life-saving services, even in times of emergency (55).

Moreover, Albania was identified as a priority country in the World Health Emergencies Programme (WHE) within the European Region World Health Organization. Each priority country, including Albania, has faced significant hazards and has exhibited vulnerabilities in health emergency response capacities during the COVID-19. The WHE Programme focuses on scaling up support to implement International Health Regulations (IHR) core capacities, aiming to yield the greatest impact in these nations in terms of health emergencies’ management (56). In response to these challenges, Albania has established a Commission on health emergency preparedness and response, with a focus on civil emergency related health aspects. This commission serves as the foundation for the development of a comprehensive emergency response system, encompassing preparedness through to recovery. In 2024, the development of the Emergency Response Plan, which will serve as the cornerstone for response’s operations to health emergency in Albania.

### 3.4 Activity volumes of the Albanian EMSs system in 2018–2021

Comparison of 2019 and 2018 data on the EMSs provided by the Centre revealed a notable increase of activity, reflecting the broader awareness and utilization of this service by the Albanian population. It’s worth noting that the Centre was implemented throughout the Albanian national territory in 2018 (see Table 1). In 2019, as compared to 2018, the following changes were observed:

- Calls to 127 increased by +99%.
- Missions ending with emergency department admission rose by +209%.
- Missions concluding on-site increased by +66%.
- Medical consultations experienced a + 71% growth.

**Table 1:** Data about Activity of National Emergency Centre 127, 2018-2021.

**Years**	**Calls to 127**		**Missions ended with emergency department admission**		**Missions ended on site**		**Medical consultations**	
	No COVID-19	COVID-19	No COVID-19	COVID-19	No COVID-19	COVID-19	No COVID-19	COVID-19
**2018**	202.349	-	19.998	-	24.464	-	22.302	-
**2019**	402.107	-	61.711	-	40.684	-	38.210	-
**2020**	557.239	190.984	61.164	7.414	59.057	8.403	82.732	92.744
**2021**	556.948	68.159	58.892	10.502	58.381	12.394	37.334	34.154

In 2019, the number of admissions to Albanian emergency departments increased by an average of +28% compared to 2018. Admissions saw a + 13% increase in district and regional hospitals and a significant +112% rise in university hospitals. Despite the implementation of pre-hospital EMSs contributing also to the consolidation of in-hospital EMSs, the percentages of admissions to emergency departments facilitated by medical transports operated by the Centre remained limited, around 4%.

**Figure 2:**
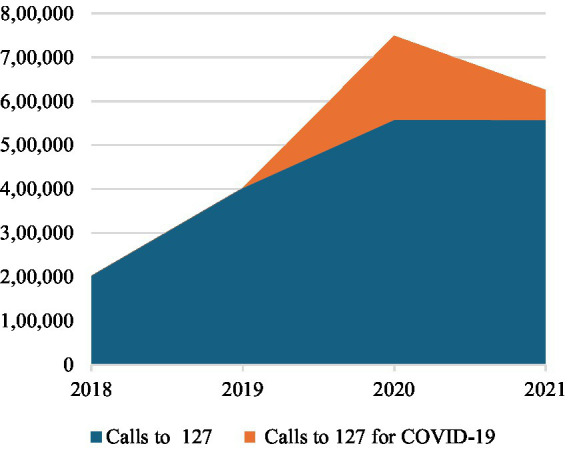
Calls to the National Emergency Centre 127, 2018-2021.

Focusing on COVID-19 pandemic period, calls grew by +86% in 2020 compared to the previous year (2019). This growth has been only +39% excluding the calls for COVID-19. Regarding the characteristic activity of the Centre, missions ending with admission in the emergency departments grew by +12% in 2019 exclusively for COVID-19; in fact, missions for others health emergencies remained essentially stable (−1%). On the other side, missions ending on-site increased +21% for COVID-19 and by +45% for others health emergencies. For this reason, access to hospital emergency departments, on average, contracted by −25% in 2020 compared to 2019. District hospitals reported a reduction in access by −19%, regional hospitals by −32%, and university hospitals by −17% in 2020 (see Table 2; Figure 2). Medical consultations experienced a significant total growth of +317%, +243% for COVID-19 and +117% for others health emergencies.

**Table 2:** Data about Hospitals’ Emergency Departments, 2019-2021.

**Years**	**District hospital**		**Regional hospital**	**University hospital**		
	**N. Admissions**	**Unit Costs**	**N. Admissions**	**Unit Costs**	**N. Admissions**	**Unit Costs**
**2019**	407.673	1568	729.040	981	364.295	1826
**2020**	331.712	2182	493.618	1309	303.825	4324
**2021**	380.301	2203	633.280	1574	351.866	2390

**Figure 3:**
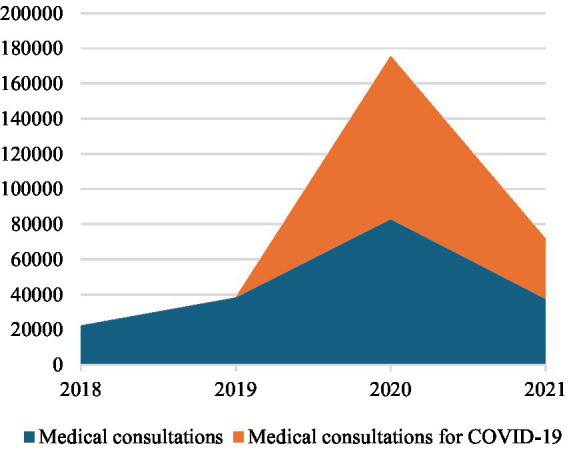
Medical Consultations by the National Emergency Centre 127, 2018-2021.

**Figure 4:**
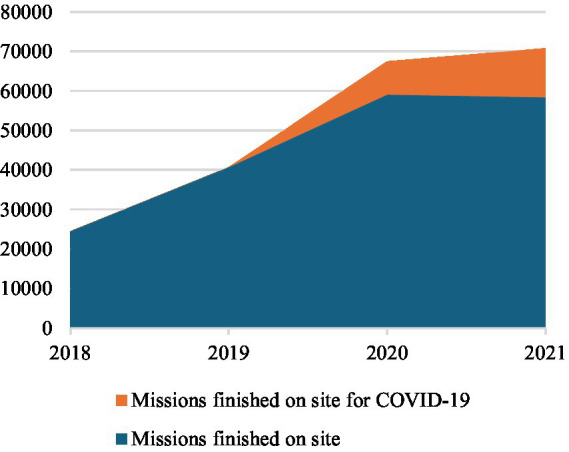
Missions Ended with Admission in Emergency Departments by the National Emergency 127, 2018-2021.

**Figure 5:**
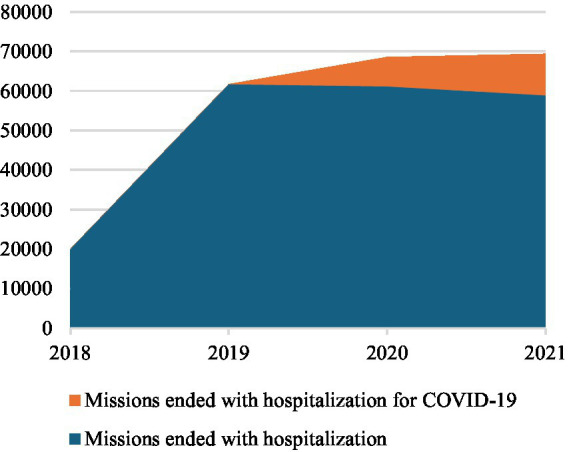
Missions Ended on Site by the National Emergency 127, 2018-2021.

**Figure 6:**
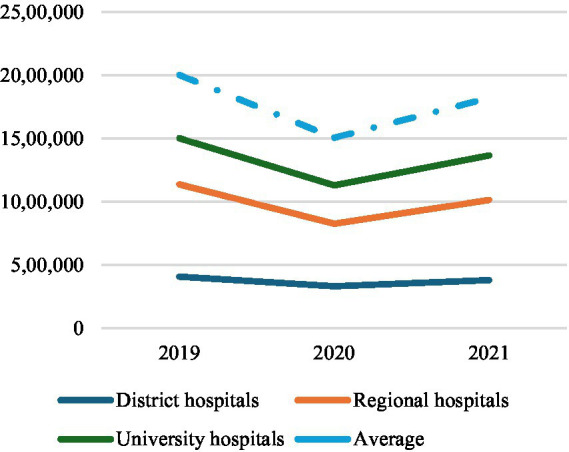
Admissions in Emergency Departments, 2019-2021.

Summarizing the commitment of the Centre for COVID-19 pandemic, in 2020 COVID-19-related calls accounted for 34% of the total calls to the Centre. Among these, 4% ended with admission to the emergency department, 4% concluded on-site, 49% involved medical consultations, and the remaining 43% were categorized as other (see Table 1; Figures 3–6).

**Figure 7:**
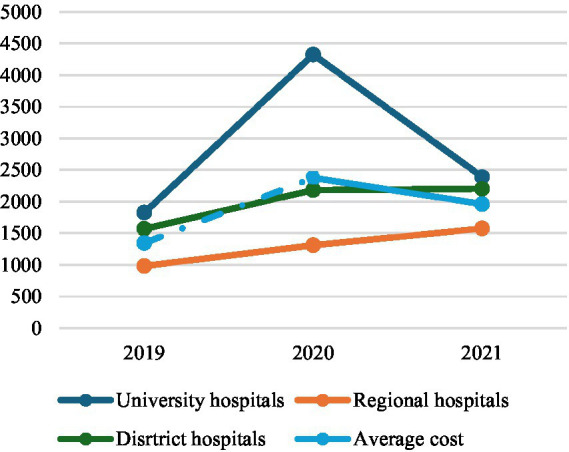
Unit cost for Admissions in Emergency Departments, 2019-2021.

In 2020, the national average cost for patient admissions to emergency departments substantially increased (77%) due to the widespread adoption of more expensive safety protocols (e.g., patient isolation, use of personal protective equipment, disinfection, surveillance) and to the efforts to limit contagion due to COVID-19 pandemic. In fact, unit costs experienced a significant increase particularly in university hospitals (137%), where COVID-19 positive patients were treated. The growth of unit costs in district and in regional hospitals was also notable with an increase of 39 and 34%, respectively (see Table 2; Figure 7). The increase of the costs was fully confirmed by the interviewees for the adoption also there of the same protocols against COVID-19 spreading considering all patients potentially COVID-19 positive (Figure 1).

In 2021 the pressure on Albanian EMSs due to the COVID-19 pandemic was relieved with a significant reduction of Centre activity compared to 2020. In fact, calls decreased by −64% due to fewer request of information about COVID-19 pandemic. Missions ending with admission of COVID-19 positive patients to emergency departments decreased by −42%, while those ending on-site by −32% due to the slowdown of the COVID-19 missions. In fact, missions were almost constant (−1% for those ending with admission to emergency departments and −4% for those ending on-site), excluding calls due to COVID-19. Medical consultations decreased by −130%, −75% for those related to COVID-19 and -55% for other health needs. In 2021, COVID-19 calls were only 12% of the total calls (15% missions ending with emergency department admission, 18% missions ending on-site, 50% medical consultation, 17% other) (see Table 1; Figures 3–6).

Accesses to hospital emergency departments, on the contrary, increased on average by +21% in 2021 compared to 2020. Emergency departments of district hospitals reported +15% of accesses, regional hospitals +28%, and university hospitals +16% (see Table 2; Figure 2). This change of strategy for COVID-19 positive patients’ hospitalization was made possible by setting up infectious disease wards within regional hospitals and equipping them with added intensive and sub-intensive care units, as confirmed by the same interviewees working here.

About costs, the shift of COVID-19 positive patients’ treatment from university to regional hospitals led to an increase in unit costs in these hospitals (20%), but it was strongly compensated by a substantial reduction in unit costs for patients assisted in university hospitals (−45%) (see Table 2; Figure 7). On the other side, at the same time, university hospitals were invited to return mainly to the treatment of ordinary health emergencies. It allowed a substantial reduction in the national average cost of each patient by −18%.

## 4 Discussion

The increasing numbers of National Emergency Centre 127 in 2018–2019 demonstrated the growing recognition of the quality and effectiveness of the offered services by this organization among the Albanian population. It is anticipated that, without considering potential health emergencies such as the COVID-19 pandemic, the numbers related to the National Emergency Centre’s activities will gradually stabilize as the entire Albanian population becomes more aware of the service’s excellence. This trend is already noticeable in the 2020–2022 period, excluding COVID-19 services.

The John Hopkins coronavirus research centre declared that Albania had 334.457 confirmed COVID-19 confirmed cases, 3.598 deaths; instead about vaccination 3,058,102 doses were administered, of which 1,345,763 (46.76% of population receiving at least 1 dose) (57).

Paying special attention to the first wave of COVID-19 pandemic in 2020, Albania was the first country in the Western Balkans to enforce a lockdown on 9th March 2020 putting in place stringent and timely measures to curb the spread and citizens’ respect of confinement orders. National government has made COVID-19 related care available to all residents and waived out-of-pocket payments, so national healthcare system was extra financed with the 4% of GDP and with external emergency financing support from international entities such as the International Monetary Fund, European Union, and other donors (58).

Additional resources were invested in the implementation of the national strategical plan against COVID-19 pandemic, in which were provided the following indications:

- The National Emergency Centre 127 was identified as reference service for the population to request information on COVID-19 pandemic and receive initial response by the Ministry of Health.
- COVID-19 positive patients in severe conditions requiring hospitalization must be assisted exclusively in the infectious disease departments of University hospitals with most of the dedicated intensive care units.

This approach reflected Albania’s first choice for a hospital-centred response to the COVID-19 pandemic, as confirmed by all interviewees.

Instead without specific guidance about pauci-symptomatic and asymptomatic COVID-19 positive patients and not complex COVID-19 negative patients, EMSs managed these cases at home in alignment with global health strategies against the COVID-19 pandemic (59) as demonstrated by increased calls to 127, medical consultations and, above all, missions ending on-site. Especially the increase of medical consultations and missions ending on-site evidenced how the implementation of pre-hospitals EMSs enlarged the response capacity of the Albania healthcare system to COVID-19 pandemic and ensured the provision of appropriate and of quality EMSs at local level limiting accesses to hospitals emergency departments and avoiding unnecessarily crowding. In this phase, district hospitals can be considered as primary healthcare centres assistance supporting the weak of community-bases health facilities in Albanian healthcare system. Emergency departments of these hospitals managed the patients with low-middle health needs, predominantly at home, for both positive (pauci-symptomatic and asymptomatic) and negative COVID-19 patients (60).

Due to that, the percentage of admissions in emergency departments in district hospitals (+2%) and university hospitals (+3%) increased, while those in regional hospitals decreased (−5%) in 2020 compared to 2019. Nevertheless, regional hospitals still accounted for 44% of the total admissions to emergency departments, district hospitals 27%, and university hospitals 24% in 2020 (see Table 2; Figure 2).

In 2020 the Albanian EMSs system resisted implementing of national strategical plan against COVID-19 pandemic. The concentration of COVID-19 positives and negatives patients in severe condition in university hospitals permitted the accumulation of experience in managing this new disease, researching, and implementing innovative treatments by practitioners who possessed the necessary knowledge and know-how. On the other side, the enforcement of local healthcare assistance for non-complex patients allowed avoiding the spread of contagion by maintaining these persons separated through domiciliary assistance (11).

In 2021, the national strategical plan against COVID-19 pandemic was updated considering increased comprehension of the disease clinical pathway (56, 61) and the discovery of COVID-19 vaccine (58, 62) as reported by all interviewees:

- The hospitalization of COVID-19 positive patients in severe condition was no exclusively in university hospitals but also in the regional ones, permitting the resumption of elective and outpatient activities.
- Formally the assistance of COVID-19 positive patients not in severe conditions was entrusted to community-based healthcare facilities and domiciliary healthcare services.

As reported in interviews, Albanian government promoted the management of all COVID-19 positive people at the local level, designating regional hospitals as reference health facilities for the treatment of symptomatic COVID-19 positives that needed hospitalization due to their severe health conditions during the pandemic second wave. Nevertheless, the Centre continued to be the reference organization in case of EMHs network for the Albanian population, even with the progressive overcoming of the COVID-19 pandemic emergency. Constant levels of ordinary pre-hospital EMSs (see Table 1; Figures 3–6) and the increasing accesses to in-hospital EMSs was especially driven by the recovery of admissions to regional hospitals due to the changed organization of Albanian in-hospital EMSs due to the update of national strategical plan against COVID-19. This rising trend was, in fact, justified by the resumption of elective activities in hospitals.

Based on the available management and economic data, along with insights gathered from interviews, the Albanian EMSs system adapted itself to the emerging evidence to struggle COVID-19 pandemic between 2020 and 2021. Albanian pre-hospital and in-hospital EMSs system collaborated to address patients’ health needs, providing the best possible response based on the organizational configuration at the time (13, 14, 38, 40, 63). Notably, there was success in strengthening the role of National Emergency Centre 127 as an information and first aid point of reference for health emergencies, aligning with the achievement not merely of the target 3.8 on universal health coverage but also of the 3.6 target on the reduction of number of global deaths and injuries from road traffic accidents and the 3.d on the strengthening of the capacity of all countries, in particular developing countries, for early warning, risk reduction and management of national and global health risks (19).

## 5 Conclusion

Albanian EMSs network demonstrated its capability to update over time the national strategical plan against COVID-19 pandemic according to emerging evidence and the related organizational issues to effectively satisfy population health needs. This adaptability became feasible with the introduction of a modern EMSs system, comprising both pre-hospital and in-hospital dimensions. These two components collaborated and are still collaborating to implement integrated healthcare pathways, each with distinct responsibilities, resources, and protocols. The development, consolidation, and collaboration between pre-hospital and in-hospital EMSs implemented in Albania have played a crucial role in preventing the collapse of the healthcare system in the face of the COVID-19 pandemic. Albanian experience provides valuable insights for the reform or to build up EMSs network and healthcare systems in transition countries, drawing upon the lessons learned from the challenges posed by the COVID-19 pandemic.

## Data Availability

The original contributions presented in the study are included in the article/Supplementary material, further inquiries can be directed to the corresponding author.
