# Correction: The emergency medical services network's response to the COVID-19 pandemic in Albania

**DOI:** 10.3389/fpubh.2025.1697485

**Published:** 2025-10-07

**Authors:** Niccolò Persiani, Martina Giusti, Francesco Taiti, Andrea Biancalani, Michele De Luca, Maria José Caldés Pinilla

**Affiliations:** ^1^Department of Experimental and Clinical Medicine, University of Florence, Florence, Italy; ^2^Centro Studi SAPIS Foundation, Italian National Federation of Orders of Radiographers and Technical, Rehabilitation, and Prevention Health Professions Research Centre, Rome, Italy; ^3^IRCCS University Children Hospital Meyer, Florence, Italy; ^4^Department of Health, Tuscany Region, Florence, Italy; ^5^Global Health Centre, Tuscany Region, Florence, Italy

**Keywords:** COVID-19, emergency medical services, assessment, health emergency strategies, transition countries

In the published article, there was an error regarding the affiliations for Niccolò Persiani and Martina Giusti. As well as having the affiliation “Department of Experimental and Clinical Medicine, University of Florence, Florence, Italy” they should also have “Centro Studi SAPIS Foundation, Italian National Federation of Orders of Radiographers and Technical, Rehabilitation, and Prevention Health Professions Research Centre, Rome, Italy”.

In the published article, there was an error regarding one of the affiliations of Michele De Luca and Maria José Caldés Pinilla. The affiliation “Centre of Global Health, Florence, Italy” should be “Global Health Centre, Tuscany Region, Florence, Italy”.

In the published article, there was an error in the article title. Instead of “Emergency healthcare services response to the COVID-19 pandemic in Albania”, it should be “The emergency medical services network's response to the COVID-19 pandemic in Albania”.

In the published article, an author name was incorrectly written as Maria Jose Caldes. The correct spelling is Maria José Caldés Pinilla.

In the published article, there was an error.

A correction has been made to **Author Contributions**

This sentence previously stated:

“NP: Conceptualization, Data curation, Formal analysis, Funding acquisition, Investigation, Methodology, Project administration, Resources, Software, Supervision, Validation, Visualization, Writing – original draft, Writing – review & editing. MG: Data curation, Formal analysis, Investigation, Writing – original draft, Writing – review & editing, Conceptualization, Methodology, Project administration, Resources, Visualization. FT: Data curation, Formal analysis, Investigation, Validation, Writing – original draft, Writing – review & editing. AB: Writing – original draft, Writing – review & editing, Data curation, Formal analysis, Investigation, Validation. ML: Conceptualization, Project administration, Resources, Supervision, Visualization, Writing – original draft, Writing – review & editing. MC: Resources, Supervision, Writing – original draft, Writing – review & editing.”

The corrected sentence appears below:

“NP: Conceptualization, Data curation, Formal analysis, Funding acquisition, Investigation, Methodology, Project administration, Resources, Software, Supervision, Validation, Visualization, Writing – original draft, Writing – review & editing. MG: Data curation, Formal analysis, Investigation, Writing – original draft, Writing – review & editing, Conceptualization, Methodology, Project administration, Resources, Visualization. FT: Data curation, Formal analysis, Investigation, Validation, Writing – original draft, Writing – review & editing. AB: Writing – original draft, Writing – review & editing, Data curation, Formal analysis, Investigation, Validation. ML: Conceptualization, Project administration, Resources, Supervision, Visualization, Writing – original draft, Writing – review & editing. MC: Resources, Supervision, Writing – original draft, Writing – review & editing.”

The original article has been updated.

