# Supplementary material for: The emergency medical services network’s response to the COVID-19 pandemic in Albania
Source: Front Public Health. 2025 Jul 30;13:1568639. doi: 10.3389/fpubh.2025.1568639 (PMC12343487; doi:10.3389/fpubh.2025.1568639)
Supplement: Supplementary file 1 [file Supplementary_file_1.docx]

**Scheme of semi-structured interview**

Section 1 - Description of health strategies.

1. Can you briefly describe the evolutive path of your organization within the Albania emergency medical services?
2. Can you briefly describe the relationship between pre-hospital and in-hospital Albanian emergency medical services?

Section 2 - Evolution of the organizational model.

1. Which were the different health strategies adopted over time by the Albanian government to manage COVID-19?
2. Can you describe the reorganization applied within your organization to address national health strategies against the first wave of the COVID-19 pandemic (2020)?
3. Can you describe the reorganization applied within your organization to address national health strategies against the next waves of the COVID-19 pandemic (2021)?

Section 3 - Consequences of health strategies.

1. Can you describe the impact due to the change of healthcare strategies adopted for the management of the COVID-19 pandemic in your organization?
